# Simulation of Spread of African Swine Fever, Including the Effects of Residues from Dead Animals

**DOI:** 10.3389/fvets.2016.00006

**Published:** 2016-02-02

**Authors:** Tariq Halasa, Anette Boklund, Anette Bøtner, Nils Toft, Hans-Hermann Thulke

**Affiliations:** ^1^National Veterinary Institute, Technical University of Denmark, Copenhagen, Denmark; ^2^Department of Ecological Modeling, Helmholtz Center for Environmental Research (UFZ), Leipzig, Germany

**Keywords:** African swine fever, model, ASF, simulation, virus

## Abstract

To study the spread of African swine fever (ASF) within a pig unit and the impact of unit size on ASF spread, a simulation model was created. In the model, an animal can be in one of the following stages: susceptible, latent, subclinical, clinical, or recovered. Animals can be infectious during the subclinical stage and are fully infectious during the clinical stage. ASF virus (ASFV) infection through residues of dead animals in the slurries was also modeled in an exponentially fading-out pattern. Low and high transmission rates for ASFV were tested in the model. Robustness analysis was carried out in order to study the impact of uncertain parameters on model predictions. The results showed that the disease may fade out within the pig unit without a major outbreak. Furthermore, they showed that spread of ASFV is dependent on the infectiousness of subclinical animals and the residues of dead animals, the transmission rate of the virus, and importantly the unit size. Moreover, increasing the duration of the latent or the subclinical stages resulted in longer time to disease fade out. The proposed model is a simple and robust tool simulating the spread of ASFV within a pig house taking into account dynamics of ASFV spread and the unit size. The tool can be implemented in simulation models of ASFV spread between herds.

## Introduction

African swine fever (ASF) is an infectious disease of pigs, caused by the ASF virus (ASFV), which is a DNA virus from the family *Asfarviridae*, genus *Asfivirus* (1). The disease has high relevance to the pig health and pig industry and is one of the most important emerging diseases of domestic pigs (2, 3). Infection with ASF is associated with a wide range of clinical symptoms from almost unapparent to severe clinical signs and death with mortality ranging from 3 to 100% (3). In the recent outbreaks observed in Eastern and Central Europe, a high mortality has occurred, in which approximately 95% of the infected animals have died following the appearance of clinical symptoms (4). The disease is endemic in Africa and is considered one of the biggest hurdles for the development of the pig sector in African countries, such as Uganda (5).

The disease has been persistent in Russia since 2008 (6), and since then it has caused many cases and outbreaks in wild boar and domestic pigs in eastern European countries, such as Poland, Estonia, Latvia, and Lithuania (4, 7). Recent studies have pointed out routes of ASF spread (8–10). Oganesyan et al. (10) pointed out the importance of anthropogenic factors for farm to farm spread of ASF and the necessity of correct implementation of biosecurity and control measures to limit the spread of ASF. Vergne et al. (8) indicated a potential spillover of infection between domestic pigs and wild boar, and recommended strict biosecurity measures and the culling of detected herds. Korennoy et al. (9) indicated that the risk of ASF spread is a function of socio-economic and geographic factors.

As discussed earlier (11, 12), there is a risk of ASF spreading further throughout Europe. An outbreak of ASF in a country with a large export of swine and swine products, such as Denmark, Germany, or the Netherlands, may have devastating economic consequences on the swine industry of that country due to export restrictions. Given the current situation of ASF in Europe, there is a need for studies that investigate the impact of control strategies to limit the spread of ASF within the European Union countries.

Simulation models have previously been widely used to study the spread of animal diseases within a country and to propose effective control strategies to limit their spread [e.g., Ref. (13–19)]. Some of the widely used between-herds spread models do not simulate disease spread within the unit of interest (the herd) mechanistically (modeling individual animals separately). This is reasonable if, e.g., for contingency planning purposes the landscape scale is of interest in the modeling and individual-based representation may overcharge technical capacities. Moreover, some of the existing model environments (e.g., NAADSM; InterSpread Plus; DTU-DADS; Be-FAST) do not allow integration of individual-based within-herd simulations in their current version, although these model environments are applied in decision support (13, 18, 20–23). On the other hand, some herd-level model environments (e.g., InterSpread Plus and NAADSM) use pre-defined cumulative probability functions to represent disease progress within the animal unit. For diseases such as foot-and-mouth disease, which can spread airborne over long distances, using the same function for all herds seems reasonable. For slower spreading diseases, such as ASF, the time period, during which the infection is present in an infectious unit (i.e., herd-level infectious period), is expected to depend on the unit size. Therefore, the infectious period may vary depending on the number of animals present. When modeling between-herd spread of ASF, the variation of infectious periods of the infectious herds may impact the risk of transmission to other herds and the probability of detection. Particularly, the spread of ASF may vary depending on the stage of infection and whether the infectious animals bled, as blood is one of the major sources for ASF spread (24, 25). Thus, it is important to propose a within-unit spread model that is capable to cover the dynamics of the disease spread within units of different size, and that can be implemented in between-herd spread models.

The objectives of this study were: (1) to propose a within-standard pig holding unit (house/barn/stable) spread model that can be applied to between-herds spread models, taking into account unit size and the epidemiological characteristics of the modeled infection; (2) to model a plausible spread of ASF within the unit given the experimental knowledge on the virus type; and (3) study the impact of unit size on disease spread.

## Materials and Methods

### Simulation Study

A dynamic Monte Carlo simulation model was developed in order to simulate the spread of ASF within a domestic pig unit (representing house/barn/stable). The discrete time step was 1 day and the model was developed using the freeware R (version 3.1.3) “smooth sidewalk” (26). The model was built and parameterized to represent the Georgian ASF strain (4, 12, 27, 28).

The numbers of units to be modeled is defined at the beginning of each run, and the numbers of animals within each unit are drawn from distributions of different unit sizes (Table 1). Animals are modeled deterministically within the unit and the model assumes random mixing of animals within each unit. The start of the infection process in each unit is assumed to be the introduction of one animal in the latent stage.

**Table 1:** **Parameters used in a model simulating within-unit spread of African swine fever in 4,000 units of varying sizes**.

Parameter	Value	Explanation and source
Probability of death following infection	0.95	Gallardo et al. (4)
The relative half-life time of ASF virus in slurries (γ)	1	Assumed to be as robust as the foot-and-mouth disease virus as derived from Bøtner and Belsham (29) and Haas et al. (30)
Maximum virus survival time in slurries (*d*_max_)	5	Davis et al. (28)
Infectiousness of residues from a dead animal relative to a clinical case (ϵ)	0–1	Not available
Infectiousness of a subclinically infected animal relative to a clinical case (μ)	0–1	Not available
Transmission rate per day (β)	0.30 or 0.60	Guinat et al. (27)
Number of animals per pig unit		Danish CHR^a^ data from 2014
Small units	2–300	The values are the minimum and the maximum limits in uniform distributions, with random selection of 1,000 pig units per category assuming all animals within a herd are located in one house (unit)
Medium size units	301–1,200	
Large units	1,201–2,250	
Very large units	2,251–10,000	

*^a^The Central Husbandry Register data provided by the Veterinary, Food and Drug Administration*.

#### Disease Stages and Distribution

**Figure 1:**
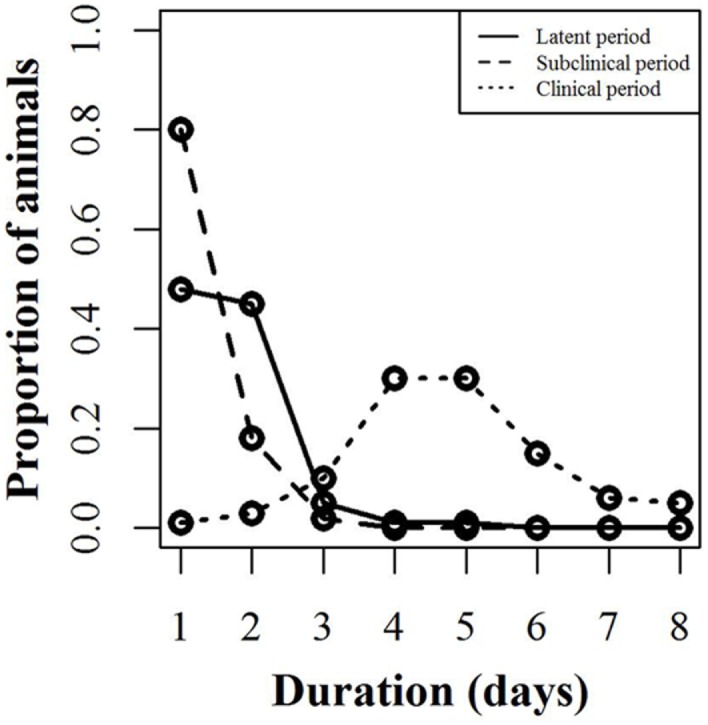
**Disease stages used for simulating African swine fever**. Proportion of animals within a pig unit (*y*-axis) that will spend the defined number of days (*x*-axis) in each infection stage (latent, subclinical, and clinical). For instance, 48% of the animals will be latent for 1 day, while 45% will be latent for 2 days.

The infection model follows the susceptible-latent-subclinical-clinical-removed (SLSCR) model. This model follows the findings from Guinat et al. (12), but with the infectious stage split into subclinical and clinical stages. This split was introduced, as it has been shown that the virus can be isolated from the blood and organs of affected pigs before clinical symptoms are observed (4). Splitting into subclinical and clinical stages facilitates the possibility of infection through subclinically infected animals and the possibility to vary infectiousness depending on factors, such as virus excretion and contact type (biting). The removed stage defines pigs that are no longer able to receive or transmit the infection. This stage includes dead animals and animals that have recovered and became immune. The distribution of the duration of the different stages is presented in Figure 1. These distributions were derived by an expert, with profound experience in ASF, based on the quantifications from Guinat et al. (12). This derivation was necessary, as the referenced study did not estimate the different stages on daily basis, nor did it split the infectious period into subclinical and clinical periods. Furthermore, experimental studies include limited numbers of animals, which limits the range of the duration of the different stages. Based on the distributions in Figure 1, the model distributes newly infected pigs to be latently infected for different time periods. For instance, 48% of the infected pigs will be latent for 1 day. Once a pig has finished the latent stage, it moves to the subclinical stage. Again, the model defines the length of the subclinical period, based on the distribution in Figure 1, and thereby, 80% of the pigs will be subclinical for 1 day, etc. After the subclinical period, pigs become clinical, and thereby infectious, and they will stay in this stage until they either die or recover and become immune. The impact of these distributions on model results was examined by changing the distribution of the latent or subclinical periods as shown in Figures S1A,B in Supplementary Material. The model parameters are summarized with their sources in Table 1.

#### Disease Spread

For each individual susceptible animal, the probability of infection (PI_t_) at each time step *t* was calculated based on the numbers of and the infectiousness of subclinical and clinical animals within the individual unit and based on the virus survival in residues from dead animals, i.e., blood, liquids, and feces, as follows:

$$PI_{\text{t}}=1-e^{\frac{-\beta *\left[({\text{Sub}}_{\text{t}-1}*\mu )+(Cl_{\text{t}-1})+\left(\varepsilon *\left(\sum_{i=1}^{\text{dm ax}}\left((e^{-(\text{i}-1)*\gamma })*{\text{dead}}_{t-i})\right)\right)\right)\right]}{N_{\text{t}-1}}}$$

where PI_t_ is the probability of infection for susceptible unit-mates at time step *t*, β is the daily transmission rate of the infection by clinical cases, with *Cl_*t*−1_* being the number of clinical cases during the previous day, *Sub_*t-1*_* is the number of subclinical cases during the previous day, μ is a parameter to address the infectiousness of a subclinical animal relative to a clinical animal, ϵ is a parameter to represent the infectiousness of the residues from a dead animal relative to a clinical animal, γ is a parameter related to the half-life of the virus in the residues. $\sum_{\text{i}=1}^{d\text{max}}\left((e^{-(\text{i}-1)*\gamma })*{\text{dead}}_{t-i}\right)$ is the contribution of the infectiousness of residues from animals that died during either of the last *d_*max*_* days prior to today; with $\left(e^{-(i-1)*\gamma }\right)$ being the infectiousness of certain residues after i days and *dead_*t*−i_* being the number of animals that were found dead at day *t* − *i*, i.e., dying *i* days prior to today. *N*_t_*_−_*_1_ is the total number of live animals within the unit during the previous day and normalizes the probability of contact between animals by unit size.

#### Transmission Parameter

From Guinat et al. (27), the transmission rates (β) within (0.6) and between (0.3) pen were used to represent a low and high transmission within the pig unit (Table 1), as no transmission rate is available measuring the joint within- and between-pen transmission within a pig unit. Therefore, we used these extreme values in two different scenarios.

#### Virus Survival in and Infection from the Environment

The model implies that the infectiousness of residues from dead animals is exponentially fading over time. Previous studies have shown that the survivability of some swine viruses (e.g., foot-and-mouth disease) in slurries fades out in an exponential pattern (29). Davis et al. (28) showed as well that the ASFV fades out exponentially in feces and urine at 21°C (survived up to 3 and 5 days, respectively). Therefore, we assume an exponential fade out of the infectiousness over time ending 5 days following the death of the animal.

The parameter γ was added to address the uncertainty of the half-life time of the ASFV, which reflects the speed of virus decay in slurry relative to virus decay in, for instance, tissue. To our knowledge, no such data are available about the currently circulating strains in Eastern Europe. Nevertheless, the data presented by Haas et al. (30) show interesting and useful results in the sense that ASF, classical swine fever, and foot-and-mouth disease virus survived in the same pattern using the same method of deactivation. Using this information and the information from Bøtner and Belsham (29), who showed that foot-and-mouth disease virus can survive in the slurries in a similar pattern, as it can in cell culture medium on a temperature of 20°C. This was also the case for ASFV in data presented by Haas et al. (30) at a temperature of 17°C during the first 2 weeks following deactivation, the parameter (γ) was set to 1.

#### Lethality

Based on Gallardo et al. (4), approximately 95% of the infected pigs would die following infection.

### Model Output, Run, and Robustness Analysis

The output of the model was the time to clearance (TTC), which represents the time between disease introduction and disease fade out within the unit. Disease fade-out was defined as neither infected animals nor infectious virus existed in the unit. The time until virus fade out would then be the time until the last animal died or recovered plus the maximum number of days the virus could survive after the death or recovery of the infected animals.

The model was run in 4,000 hypothetic units of varying size. Initial runs showed that this number of units would be sufficient to cover stochastic variability. Pig units were categorized in four hypothetical size categories (Table 1), in order to represent structural variations in housings of domestic pigs. For each category, disease spread was simulated in 1,000 hypothetic units, and the size of each unit was selected from a uniform distribution described by the minimum and maximum numbers of animals in the unit (Table 1). In the model simulations, the unreferenced parameters ϵ and μ were varied systematically from 0 to 1, in steps of 0.1 (i.e., 11 values of ϵ combined with 11 values of μ, leading to 121 different combinations).

### Statistical Analyses

The correlation between unit size and TTC was tested using Spearman correlation coefficient, because the data were not normally distributed. The difference in TTC between the different unit size categories was tested for statistical significance using the Kruskal–Wallis Test (kruskal.test function) in R (26).

## Results

### Descriptive Results

The median value of incubation period (time between infection and appearance of clinical signs) was 4 days and varied with 5th and 95th percentiles of 3 and 5 days, respectively, for the different values of ϵ, μ, and β. Table 2 shows the proportion of units with major epidemics (at least 50% of the animals were infected) for different values of ϵ and μ and assuming low and high virus transmission. For instance, with low transmission and when ϵ and μ were both 0, about 49% of the units are predicted to experience major epidemics before the disease fades out. Furthermore, when the values of ϵ and/or μ were increased, the proportion of units with major epidemics increased. For the same values of ϵ and μ, but with a high virus transmission, the proportion of units with major epidemics was 90% (Table 2). Table 2 also shows that with a high transmission, regardless of the value of ϵ and μ, the vast majority of the units will experience major epidemics following introduction of the virus.

**Table 2:** **Results of the within-herd outbreak simulation**.

Value of **β**	Value of **μ**	Value of **ϵ**	Proportion of major outbreaks^a^
0.3	0	0	0.49
	0	0.5	0.64
	0	1	0.74
	0.1	0.1	0.57
	0.5	0	0.61
	0.5	0.5	0.72
	1	0	0.71
	1	1	0.83
0.6	0	0	0.90
	0	0.5	0.94
	0	1	0.96
	0.1	0.1	0.92
	0.5	0	0.93
	0.5	0.5	0.96
	1	0	0.95
	1	1	0.98

*Depending on the values of the infectiousness parameters μ and ϵ, we show the proportion of major outbreaks for low (0.3) and high (0.6) transmission rate (β)*.

*^a^Proportion of herds where >50% of the animals within the herds were infected following the introduction of infection to the herd*.

**Figure 2:**
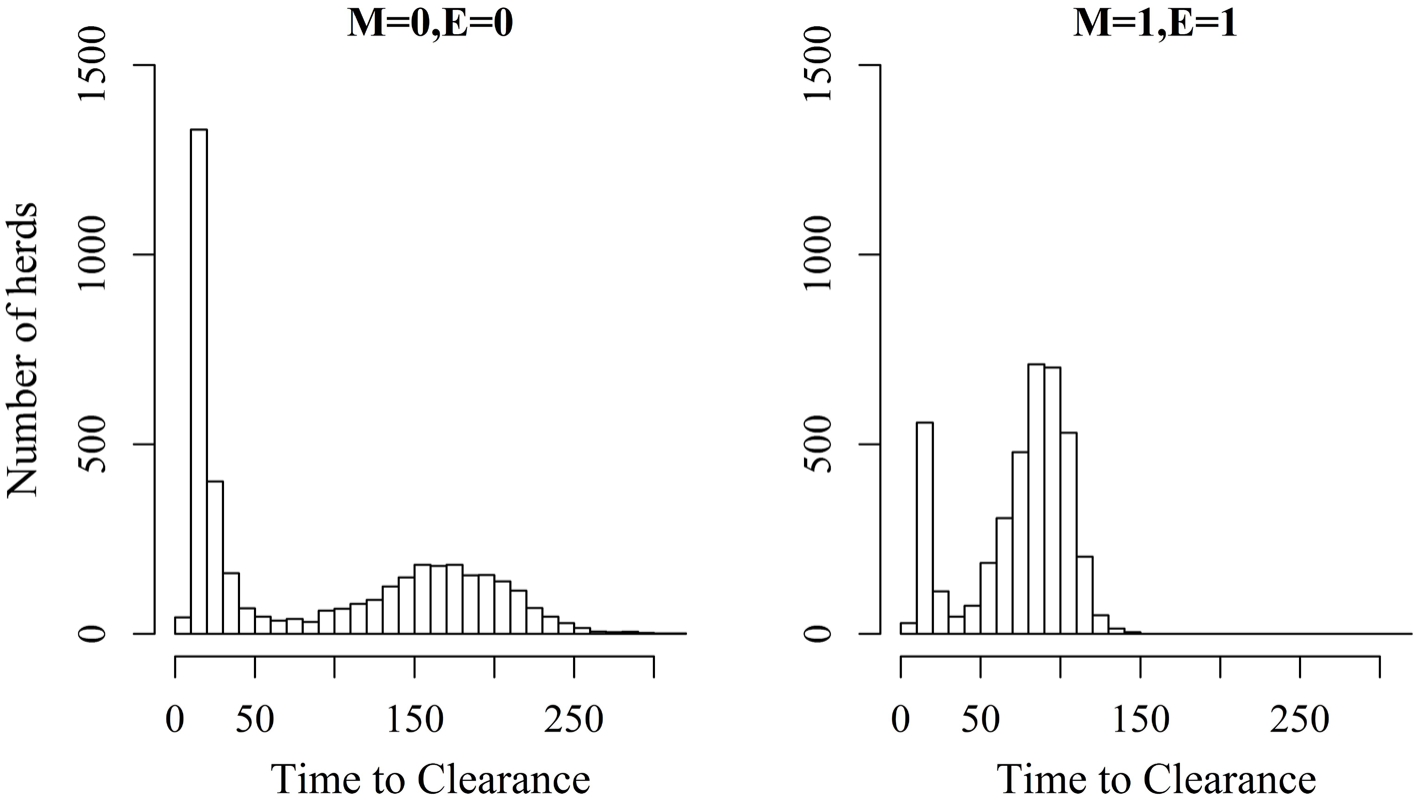
**Distribution of the time it takes until African swine fever has died off or infected all animals in a domestic pig unit [time to clearance (TTC)] for different values of μ(M) and ϵ (E) at a low virus transmission rate (β = 0.3)**.

**Figure 3:**
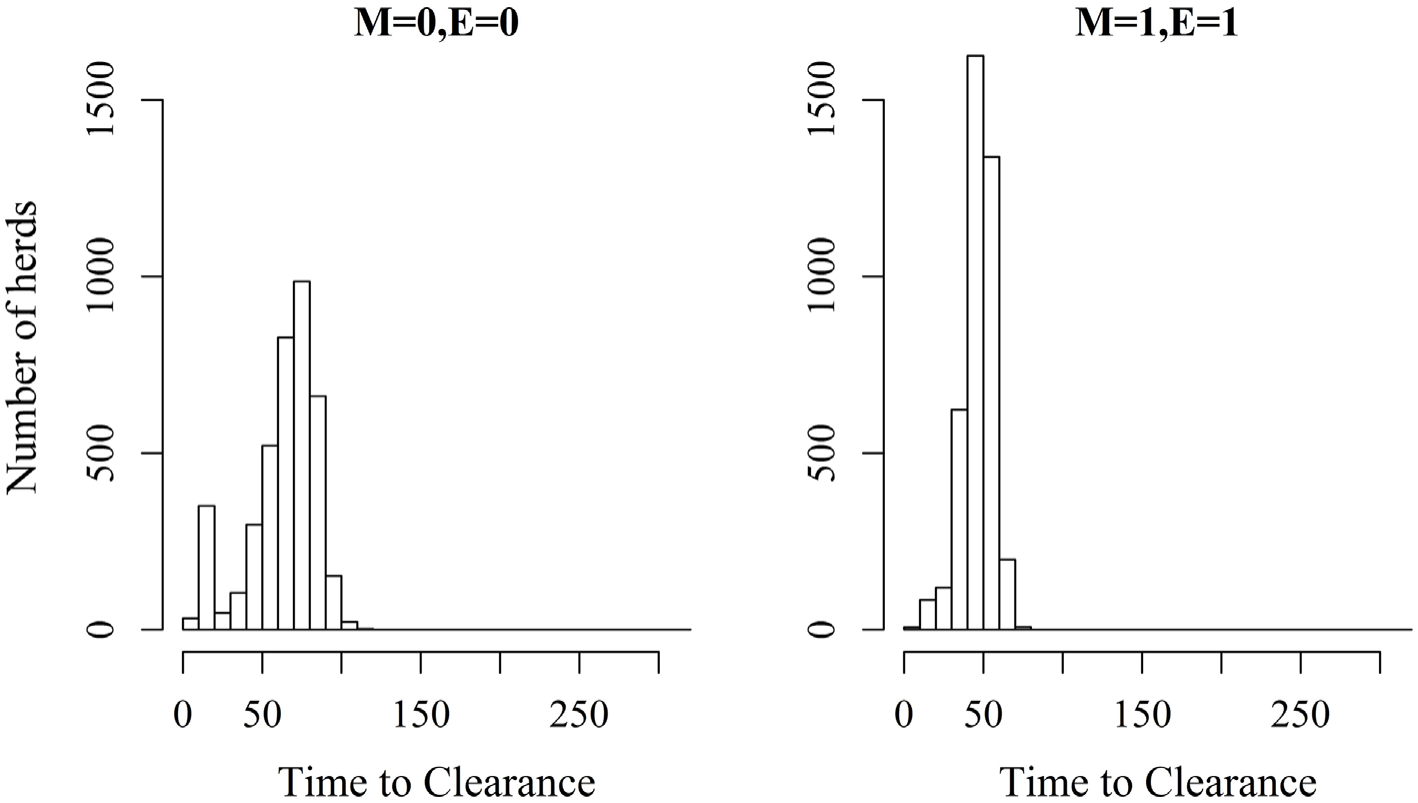
**Distribution of the time it takes until African swine fever has died off or infected all animals in a domestic pig unit [time to clearance (TTC)] for different values of μ (M) and ϵ (E) at a high virus transmission rate (β = 0.6)**.

Figures 2 and 3 show the unit-frequency of TTC for selected values of ϵ and μ when low (high) virus transmission is modeled (more data are presented in Figures S2A,B in Supplementary Material). These figures translate Table 2 into time. The majority of outbreaks that did not end up in a major epidemic were finished within 20 days corresponding to the sum of the disease stage periods. For instance, when ϵ and μ were both 0, the disease died out in 11% (3%) of the epidemics without spreading to other animals within the unit. Both figures show that increasing the values of ϵ and/or μ resulted of course in shorter TTC both with low and high virus transmission. This is due to the increased probability of disease spread, when ϵ and/or μ values are increased. On the other hand, TTC seems to vary more when a low transmission rate is modeled, compared to a high transmission rate. Furthermore, when high transmission rate is modeled, TTC is of course much shorter than when low transmission is modeled. It is important to mention that the variation in these figures is due to unit size.

**Figure 4:**
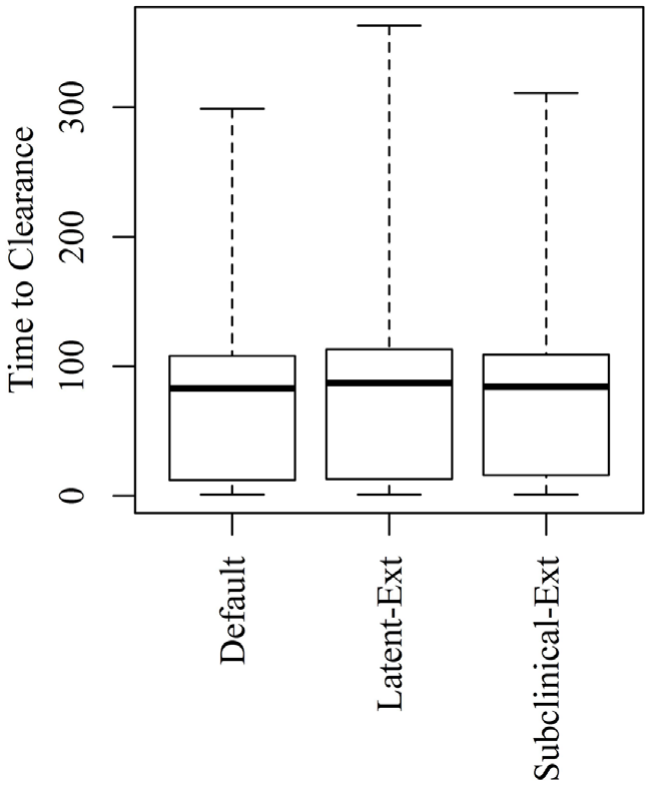
**Time to clearance (TTC) following changing the distributions of the latent (Latent-Ext) or the subclinical (Subcinical-Ext) periods compared to the default distributions (Default) under low virus transmission rate (β = 0.3) and for all combinations of μ and ϵ**.

**Figure 5:**
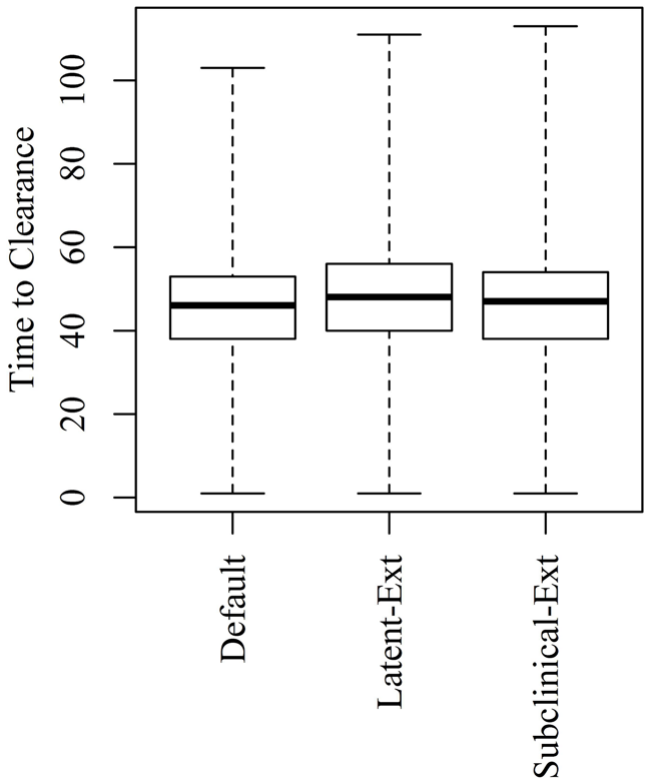
**Time to clearance (TTC) following changing the distributions of the latent (Latent-Ext) or the subclinical (Subcinical-Ext) periods compared to the default distributions (Default) under high virus transmission rate (β = 0.6) and for all combinations of μ and ϵ**.

Extending the latent or the subclinical stages (Figures S1A,B in Supplementary Material) would result in only a slight increase to the TTC compared to the default distribution presented in Figure 1 using all combinations of μ and ϵ at low and high virus transmission rates (Figures 4 and 5).

### Impact of Unit Size on TTC

**Figure 6:**
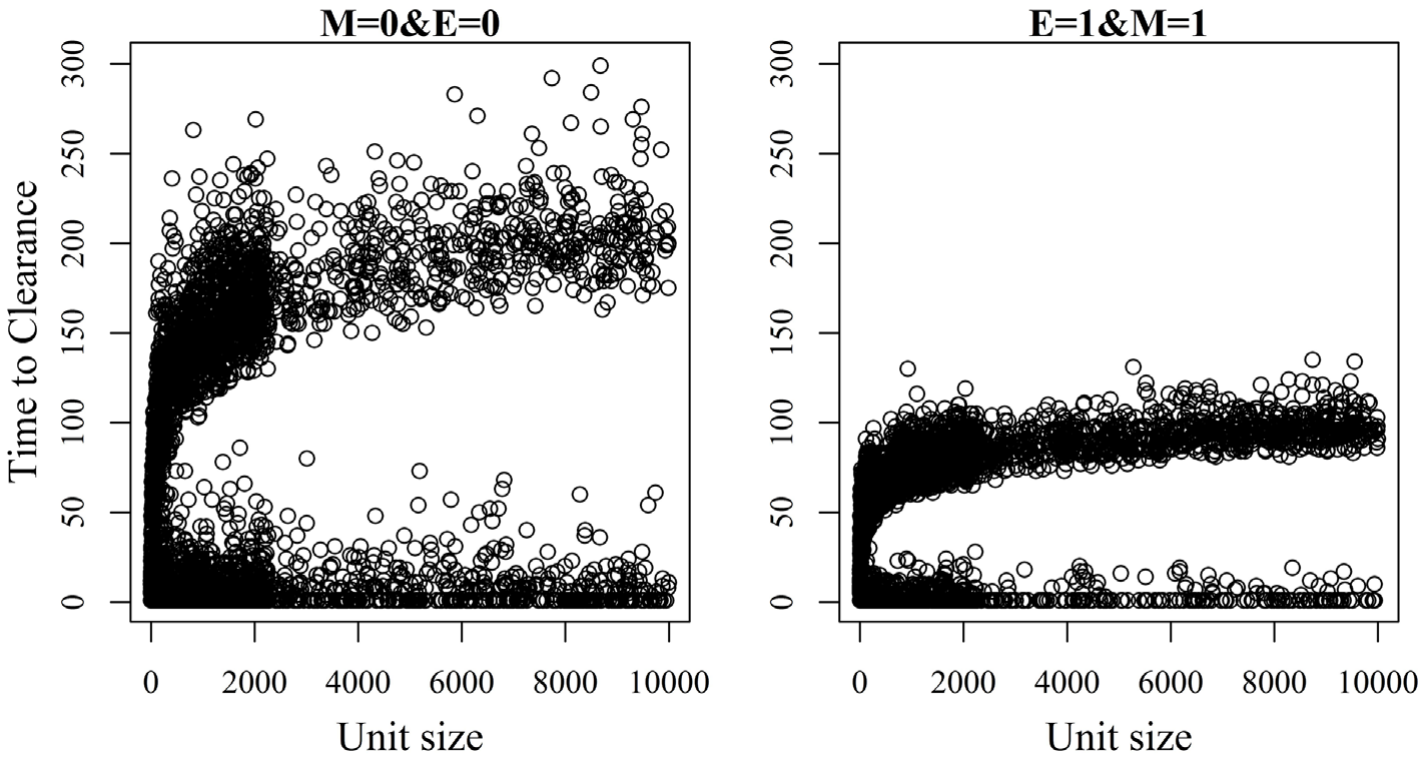
**Association between unit size and the time until African swine fever has either died off or all animals has become infected [time to clearance (TTC)] for different values of μ (M) and ϵ (E), and with a low virus transmission rate (β = 0.3)**.

**Figure 7:**
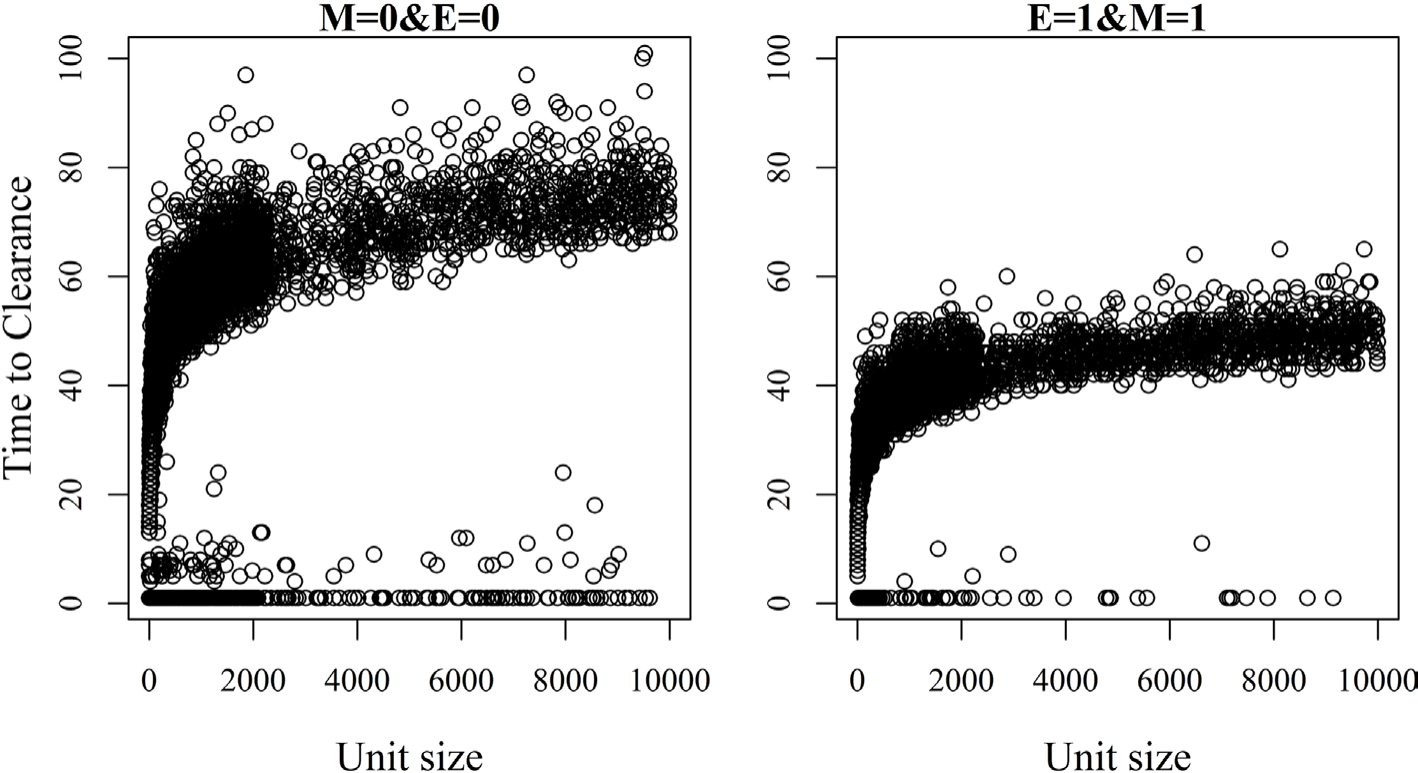
**Association between unit size and the time until African swine fever has either died off or all animals has become infected [time to clearance (TTC)] for different values of μ (M) and ϵ (E), and with a high virus transmission rate (β = 0.6)**.

The relationship between unit size and TTC for selected values of ϵ and μ and under a low (high) transmission rate is shown in Figures 6 and 7. More data are presented in Figures S3A,B in Supplementary Material. Under low virus transmission rate and in units with TTC > 50, the correlation between unit size and TTC varied between 0.80 and 0.85 for all the different combinations of ϵ and μ. This shows that unit size had a large impact on TTC, regardless of the values of ϵ and μ. When a high transmission rate was modeled (Figure 7 and Figure S3B in Supplementary Material), the correlation between unit size and TTC varied between 0.71 and 0.87 for the different combinations of ϵ and μ, and the correlation increased with increasing values of ϵ and/or μ. In units with TTC > 20 days, the correlation varied between 0.87 and 0.89 for the different combinations of ϵ and μ. In all combinations of ϵ and μ and for the low and high transmission rates, the correlation coefficients were statistically significant (*P*-values <0.001). Furthermore, there was statistically significant difference in TTC between the different unit size categories (categories are based on distributions presented in Table 1) for all combinations of ϵ and μ and under the low and high virus transmission (*P*-values <0.001). Naturally, the larger the unit size is, the longer the TTC is.

**Figure 8:**
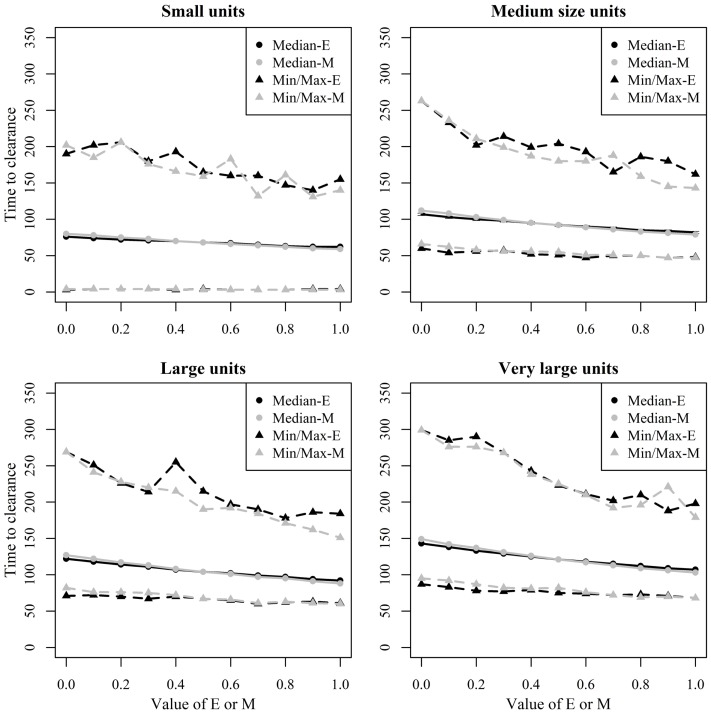
**Time until African swine fever would either died off or all animals would be infected [time to clearance (TTC)] for different domestic pig unit size categories and different values of μ (M) and ϵ (E) at a low virus transmission (β = 0.3) for major outbreaks, in which at least 50% of the animals within the unit were infected**. TTC estimates for all μ values (from 0 to 1) were summarized for each value of ϵ. Similarly, TTC estimates for all ϵ values (from 0 to 1) were also summarized each value of μ.

**Figure 9:**
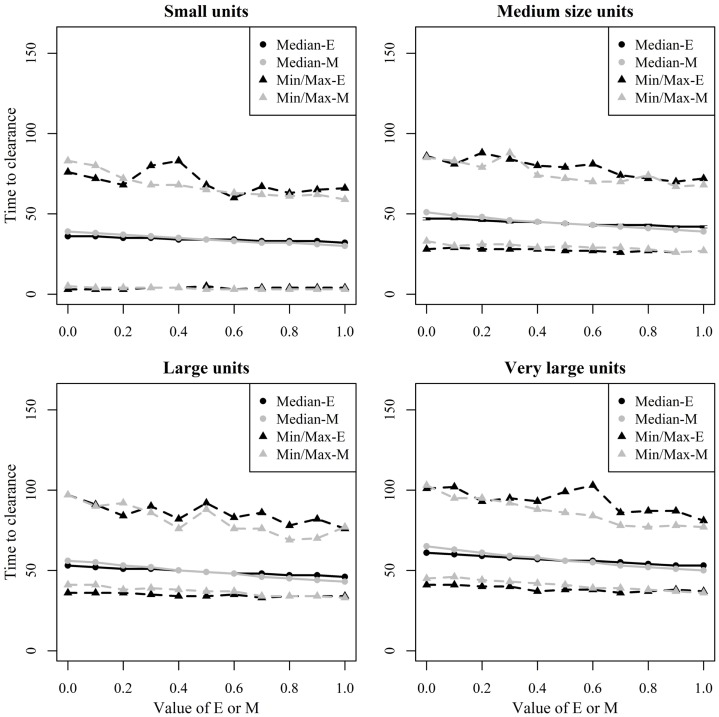
**Time until African swine fever would either died off or all animals would be infected [time to clearance (TTC)] for different domestic pig unit size categories and different values of μ (M) and ϵ (E) at a high virus transmission (β = 0.6) for major outbreaks, in which at least 50% of the animals within the herds were infected**. TTC estimates for all μ values (from 0 to 1) were summarized for each value of ϵ. Similarly, TTC estimates for all ϵ values (from 0 to 1) were also summarized each value of μ.

Figure 8 shows the median and range values of TTC for different values of ϵ and μ and different unit size categories with a low transmission rate for units where at least 50% of the animals were infected. Figure 9 shows the same relationship but with a high transmission rate. The two figures clearly show that the transmission rate parameter has substantial impact on TTC. Furthermore, the impact of ϵ, μ, and unit size is consistent with low and high transmission.

## Discussion

This study simulates ASFV spread between animals within a pig unit (house/barn/stable). The model has the advantage that it takes into account unit size and important factors on animal level, such as duration of the different disease stage periods, despite that it does not model individual animals mechanistically. The model considers the different stages of disease, which contributes to the transmission dynamics (e.g., subclinical, clinical, and dead animals). Moreover, latency period was considered following the results of Guinat et al. (12); while Gallardo et al. (4) indicated that the virus was detected in the blood by PCR prior to the appearance of clinical signs. This indicates that the animals maybe infectious prior to clinical symptom appearance (subclinical period). Pigs may be detected in the subclinical stage, as the virus is shed in the blood, despite that they may not be infectious. This is important for modeling surveillance by blood samples when modeling disease spread between herds. The model also addresses the potential spread of the virus through residues from dead animals in slurries. The ASFV is a robust virus that is capable of surviving in organic materials for some days (28, 31). Although infection leads to the death of most of the affected animals [approximately 95% according to Gallardo et al. (4)], and hence a limiting factor for disease spread, virus in blood, feces, and liquids from infected animals can persist in the slurry following biting by other pigs or from hemorrhagic diarrhea (4), and hence be a potential risk for disease spread.

The results show that TTC varies depending on ϵ and μ. The infectious potential of subclinical animals (μ) is unknown, despite that scientific evidence has shown that animals may have viremia before clinical symptoms appear, as discussed above (4), indicating the potential of virus spread from these animals. From a practical standpoint, it is possible that subclinically infected pigs have scratches or small bites, which might lead to access to blood for the pen mates, and hence lead to disease spread. Nevertheless, this theory needs testing. However, for the predictive TTC quantification, this possible extra time of infectiousness had limited influence.

As recent work has shown that infectious virus was isolated from feces and urine up to 5 days after storage (28), it was important to model virus survivability in the slurry and the potential spread of virus through this path. Nevertheless, from practical standpoint, despite that infectious virus exists in the slurry, it is unknown how much it may contribute to disease spread and, thus, the parameter ϵ was added. The results show that ϵ has a considerable impact on the results, indicating the importance of quantifying it in an experimental framework. This could be done experimentally by, for instance, introducing susceptible pigs to an area where infectious pigs had been staying. Although less relevant for the TTC quantification, ϵ might have considerable impact on the risk of ASF transmission out of an infected unit.

Changing the distribution of the latent or subclinical periods to increase the probability that animals having longer latent or subclinical periods resulted in a slight increase to the median TTC using both low and high transmission rates (Figures 4 and 5). Samples are usually not collected on daily basis from animals during the experiments, perhaps due to ethical reasons. This makes it difficult to extract exact durations of these stages and, hence, can create variability in model outcomes. Thus, accurate assessment of the duration of these stages is necessary for accurate simulation of ASF spread.

Unit size has a significant impact on disease spread within a unit (Figures 8 and 9). This clearly shows the importance of considering the number of animals; when simulating ASF spread between herds. It is clear that a contact infection spreading through a unit will take longer, the larger the house population is. However, for the purpose of developing models useful to support control planning, it was important to understand what impact different unit sizes may have on TTC compared to other debated aspects, likewise the infectivity of subclinical cases or carcasses residues. Thus, when simulating the spread of ASF between herds, the use of pre-defined cumulative probability functions that result in a similar disease progress pattern regardless the unit size may mis-estimate the infectiousness of the infected herds, which may result in inappropriate disease spread between herds.

The TTC changed dramatically under low and high transmission with similar ϵ and μ values (Figures 8 and 9). According to Guinat et al. (27), virus transmission rates for within- and between-pen transmission were 0.6 and 0.3, respectively, assuming a latent period of 4 days. The information provided by Guinat et al. (27) is inconclusive concerning the magnitude of the potential difference between both transmission rates due to the strong overlap of the confidence intervals of both parameters. Thus, a more sophisticated approach that disentangles within- and between-pen spread mechanistically is of low value here. Basically, any supposedly assumed difference between the transmission within or between pens would multiply with the size of the unit, thus, bringing not much greater precision regarding the comparative assessment of ϵ, μ, and unit size on TTC in the maximum range spanned by the average β within or between pens. Pietschmann et al. (25) estimated the reproduction number (*R*_0_) of within pen transmission of ASFV (the Caucasian strain) for domestic pigs, and wild boar to be 6.1 (95% confidence interval of 0.6–14.5) and 5 (95% confidence interval of 1.4–10.6), respectively. The between-pen *R*_0_ was estimated to be 0.5 (95% confidence interval of 0.1–1.3). In comparison, the *R*_0_ corresponding to the β-estimates of Guinat et al. (27) are 2.8 (1.3–4.8) within and 1.4 (0.6–2.4) between pens. Thus, the knowledge on ASF transmission parameters available from animal experiments is associated with rather huge uncertainty. In order to improve precision of predictive ASF modeling within herds, the more precise estimation of the transmission parameters will provide the greatest improvement, compared to the impact of chosen ϵ and μ values. Most important here, the impact of μ, ϵ, and unit size was consistent for the wide range of β.

de Carvalho Ferreira et al. (1) estimated the transmission rate of the Malta78 and Netherlands86 strains of ASFV based on detection through RT-PCR in OPF fluid or 1 or 2 days following occurrence of pyrexia. When detection based on PCR was the criteria, the transmission rates were considerably higher than those estimated by Guinat et al. (27) for the Georgian strain. However, when estimated based on onset of pyrexia, the transmission rates were close to those estimated by Guinat et al. (27). In the simulation study of Guinat et al. (27), the probability of outbreak failure (disease does not spread to other animals within the unit) varied between 7 and 22%, and the probability of not all animals in the unit become infected varied between 10 and 65% depending on the duration of the latent period. In our simulations, the disease faded out in 1–11% of the units and the probability of not all animals in the unit become infected varied between 6 and 99%, depending on the values of β, ϵ, and μ, before spreading to other animals. From the eastern European epidemics, it has been estimated that ASF outbreaks could go extinct within a pig unit with 10–17% probability and small-scale epidemics were observed in 18–45% of the epidemics (11, 27). In our simulations, small-scale epidemics (at most 10% of the animals within the unit were infected) were observed in approximately 2–50% of the simulated units, depending on the values of β, ϵ, and μ. Our results overlap with results from Guinat et al. (27) and the field observations, but seem to have more extreme values. The overlap between our results and Guinat et al. (27) is not surprising as we used their data to parameterize our model. On the other hand, the differences can be explained by the fact that we use extreme values of transmission rates and that we use an extra compartment (subclinical stage). Furthermore, Guinat et al. (27) did not model the potential spread of the virus from residues of dead animals in the slurries.

Backer et al. (32) simulated the spread of classical swine fever in the Netherlands on pen level and assuming 10 animals within a pen. The same model structure was used to simulate the spread of swine vesicular disease (33). However, these models still assumed random mixing between pens, and assumed that all animals reside within one unit, as transmission between units was not modeled. Given the inconclusive results regarding the difference between within- and between-pen transmission and the available evidence of the possibility of airborne spread of ASFV within a pig unit (34), the random mixing assumption within a unit may be acceptable. Thus, the current model can be a reasonable tool to simulate the spread of ASFV within a pig unit, given the restrictions. Nevertheless, when simulating the spread of infection between herds, information about the number of units in very large herds may be necessary to obtain. Because of the normally high biosecurity in these herds, the transmission between units is expected to be slower than the transmission within a unit, and hence may be important to simulate. Still data about the number of units per herd might be difficult to obtain as such data are not normally available in the national registers, as in the case of Denmark.

Several widely used models, such as the InterSpread Plus, NAADSM, Be-FAST, and DTU-DADS, use detailed data on movements and contacts to simulate disease spread on the regional scale (13, 18, 20–23). This limits their capacity to simulate within house transmission on individual animal level mechanistically considering penning and sectioning within the herd. Therefore, the main objective of the study was to provide a within-unit transmission model, which does not require mechanistic modeling of individual animals, but, at the same time, does consider the different stages of the disease for the individual animals deterministically, given the available experimental evidence of the disease stage periods and transmission parameter values on animal level. It is inconclusive in the literature, whether or not, or how much, the different potentially infectious stages (subclinical, clinical, or dead) contribute to the transmission of the virus. To understand the expected time ASFV will circulate in a pig herd, we found it useful to test the effect of either of the infectious stages on the time till fade-off of ASF in infected units. The proposed model can be implemented in between-herds spread model without inflating the running time of the models, while considering important factors that affects disease spread, such as infection stages on the individual animal level and unit size.

## Conclusion

The presented model is a robust tool simulating the spread of ASF within a pig unit taking into account dynamics of ASF spread and unit size. The tool can be implemented in models simulating spread of ASF between herds. The larger the unit size is, the longer the time until the disease has faded out or all animals are infected and, hence, unit size should be considered, when spread models of ASF are developed. Experimental studies are needed to quantify important parameters for ASF spread within a herd, including the transmission rate, the infectiousness of subclinically infected animals, and the infectiousness of residues from dead animals in slurries.

## Author Contributions

All authors participated in study design and results discussions. TH and H-HT developed the model. TH wrote the manuscript.

## Conflict of Interest Statement

The authors declare that the research was conducted in the absence of any commercial or financial relationships that could be construed as a potential conflict of interest.
