# Supplementary material for: Simulation of Spread of African Swine Fever, Including the Effects of Residues from Dead Animals
Source: Front Vet Sci. 2016 Feb 2;3:6. doi: 10.3389/fvets.2016.00006 (PMC4735426; doi:10.3389/fvets.2016.00006)
Supplement: Supplementary file 1 [file data_sheet_1.docx]

Supplementary Material

Simulation of spread of African swine fever, including the effects of residues from dead animals

**Tariq Halasa^1*^, Anette Boklund^1^, Anette Bøtner^1^, Nils Toft^1^, Hans-Hermann Thulke^2^**

*** Correspondence:** Corresponding Author: tahbh@vet.dtu.dk

# Supplementary Figures and Tables

## Supplementary Figures


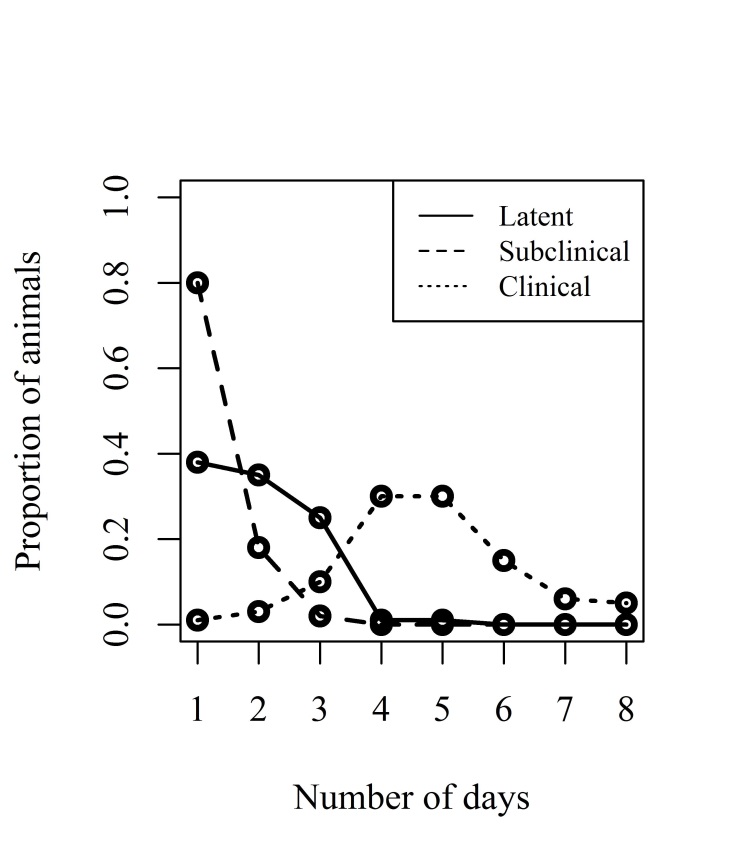


**Supplementary Figure S1a**. Disease stages used for simulating African swine fever. Proportion of animals within a pig unit (y-axis) that will spend the defined number of days (x-axis) in each infection stage (latent, subclinical and clinical). The latent stage was prolonged in order to study its impact on model outcomes.


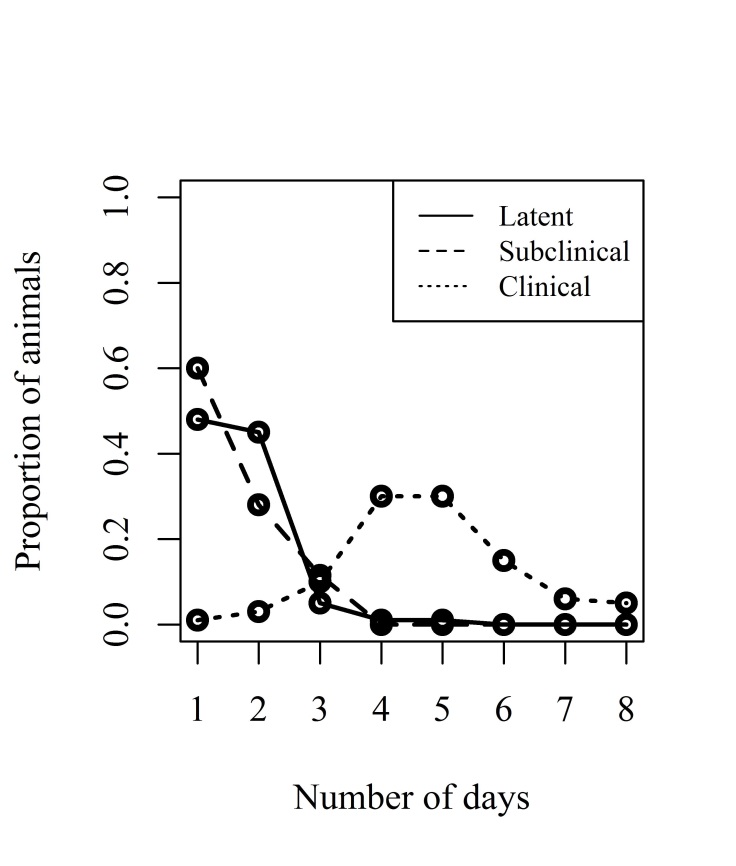


**Supplementary Figure S1b**. Disease stages used for simulating African swine fever. Proportion of animals within a pig unit (y-axis) that will spend the defined number of days (x-axis) in each infection stage (latent, subclinical and clinical). The subclinical stage was prolonged in order to study its impact on model outcomes.


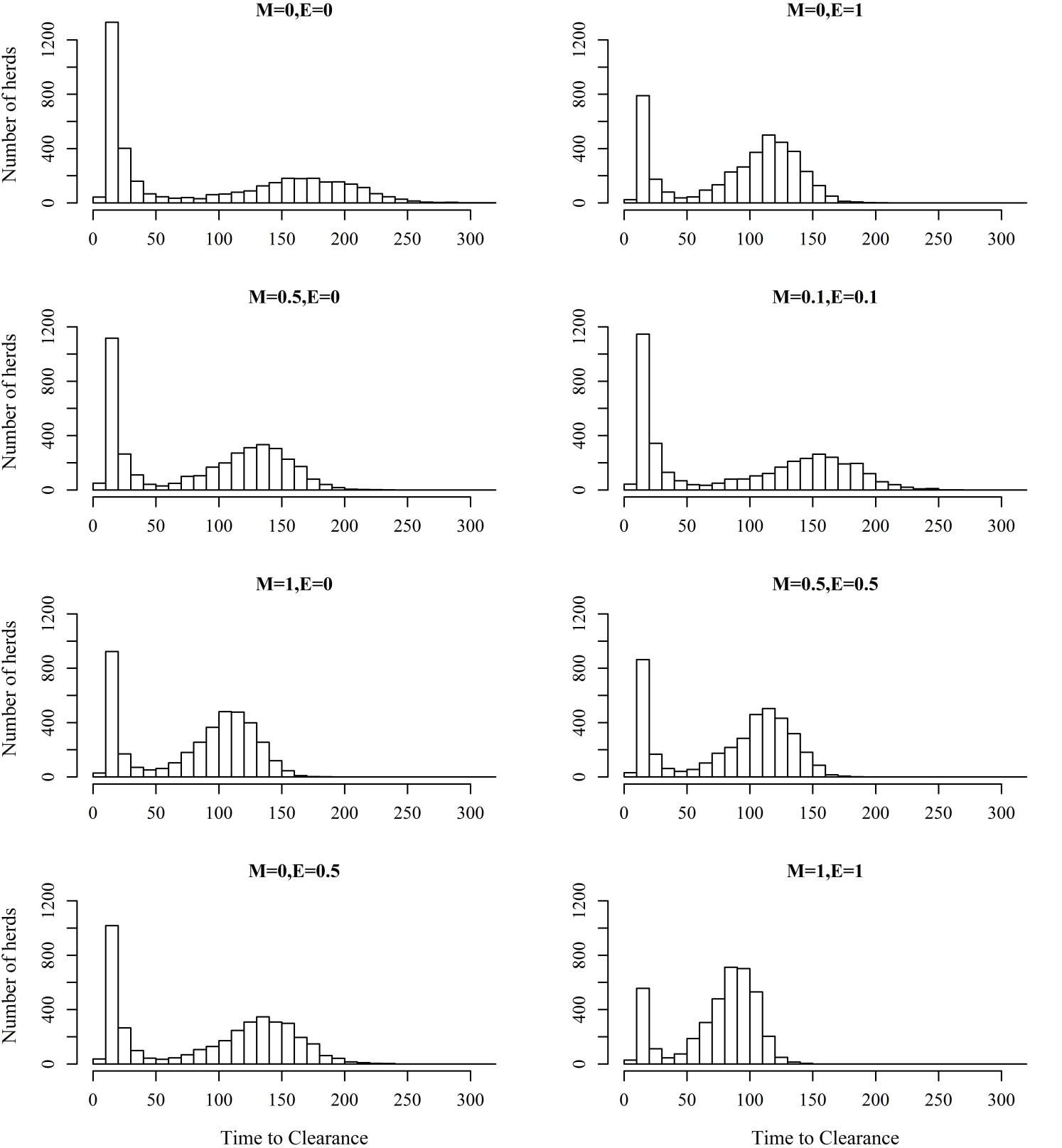


**Supplementary Figure S2a.** Distribution of the time it takes until African swine fever has died off or infected all animals in a domestic pig unit (time to clearance - TTC) for different values of μ(M) and ε (E) at a low virus transmission rate (β = 0.3)


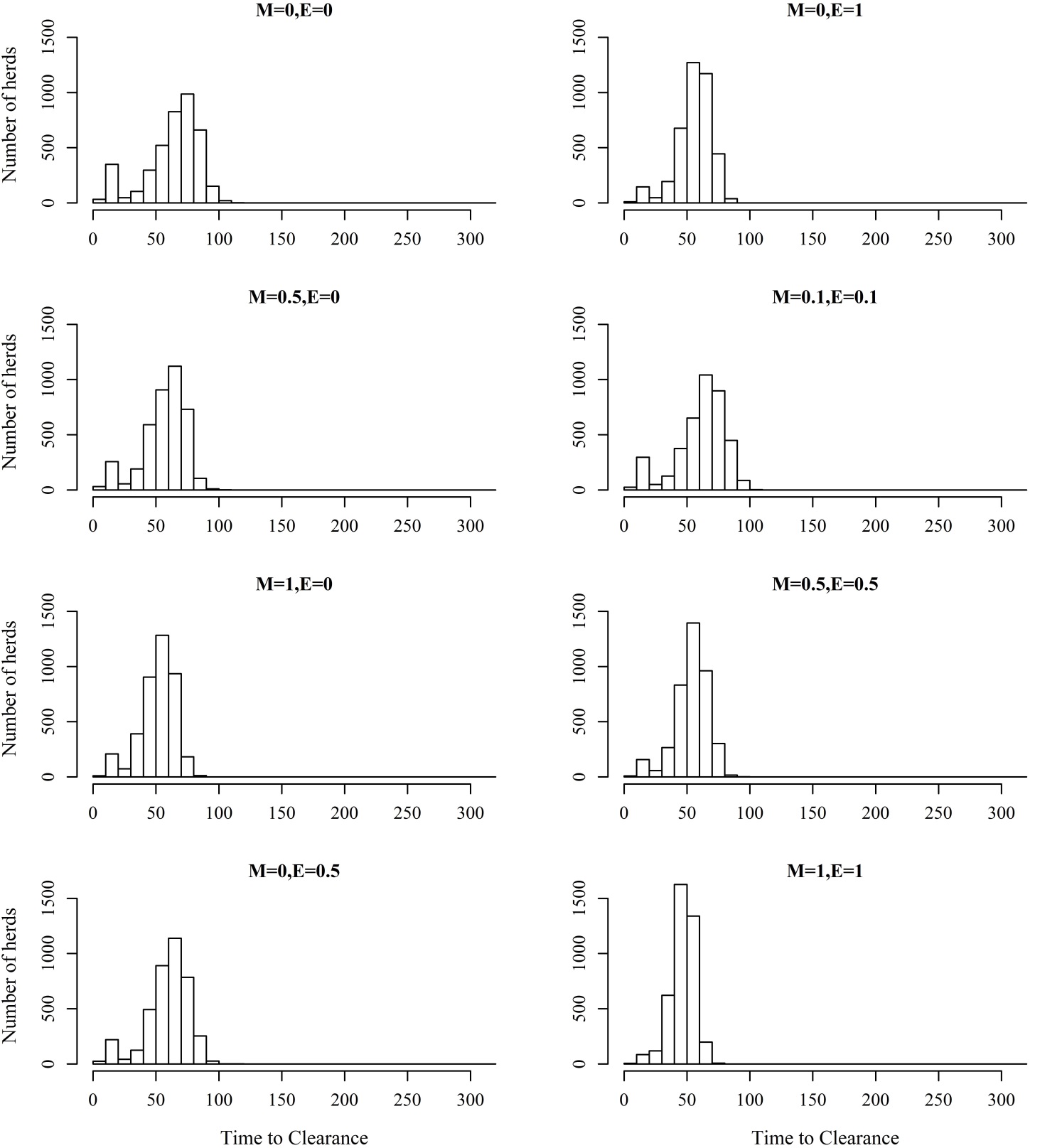


**Supplementary Figure S2b.** Distribution of the time it takes until African swine fever has died off or infected all animals in a domestic pig unit (time to clearance - TTC) for different values of μ (M) and ε (E) at a high virus transmission rate (β = 0.6).

**
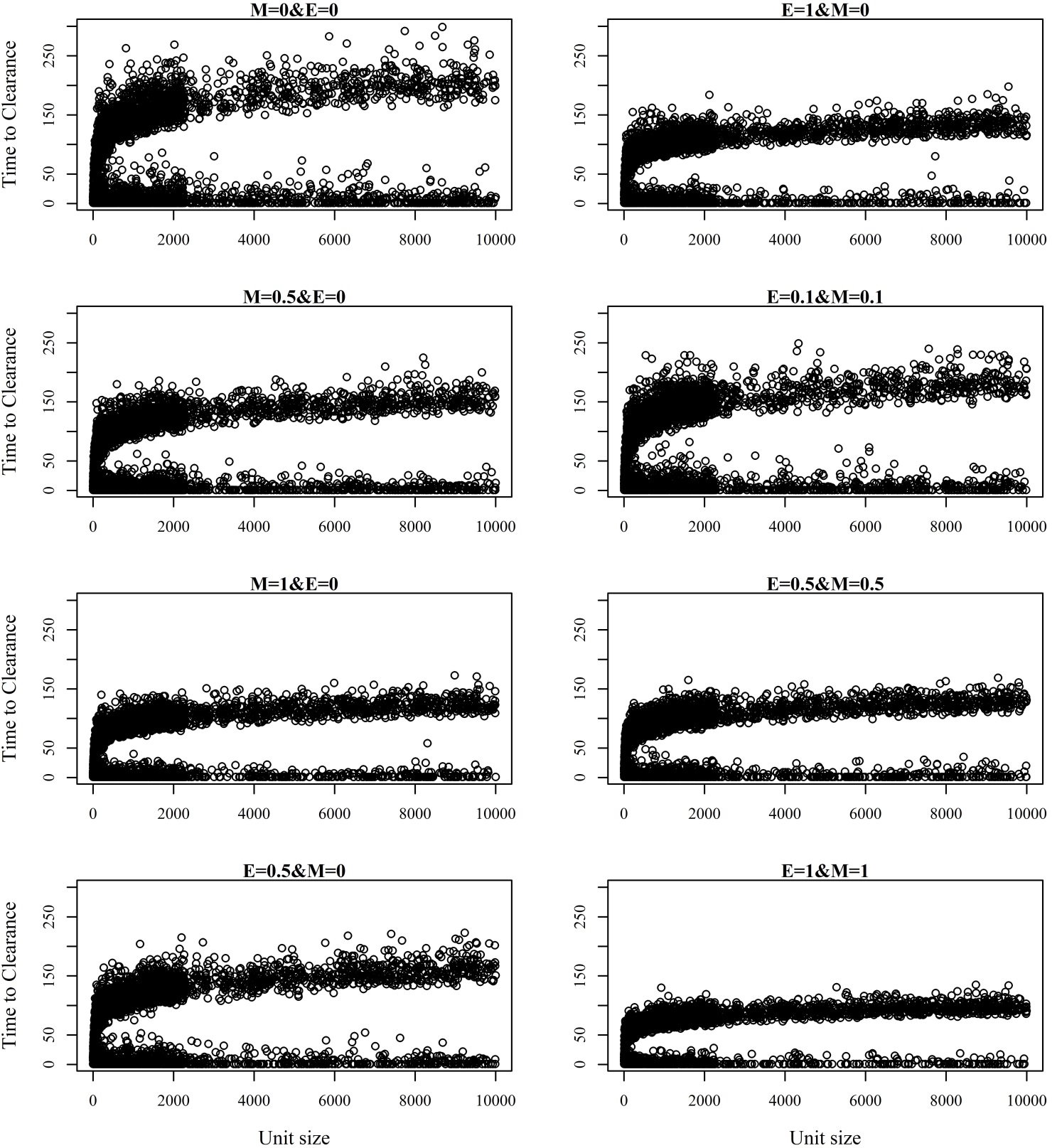
**

**Supplementary Figure S3a.** Association between unit size and the time until African swine fever has either died off or all animals has become infected (time to clearance - TTC) for different values μ (M) and ε (E), and with a low virus transmission rate (β = 0.3).


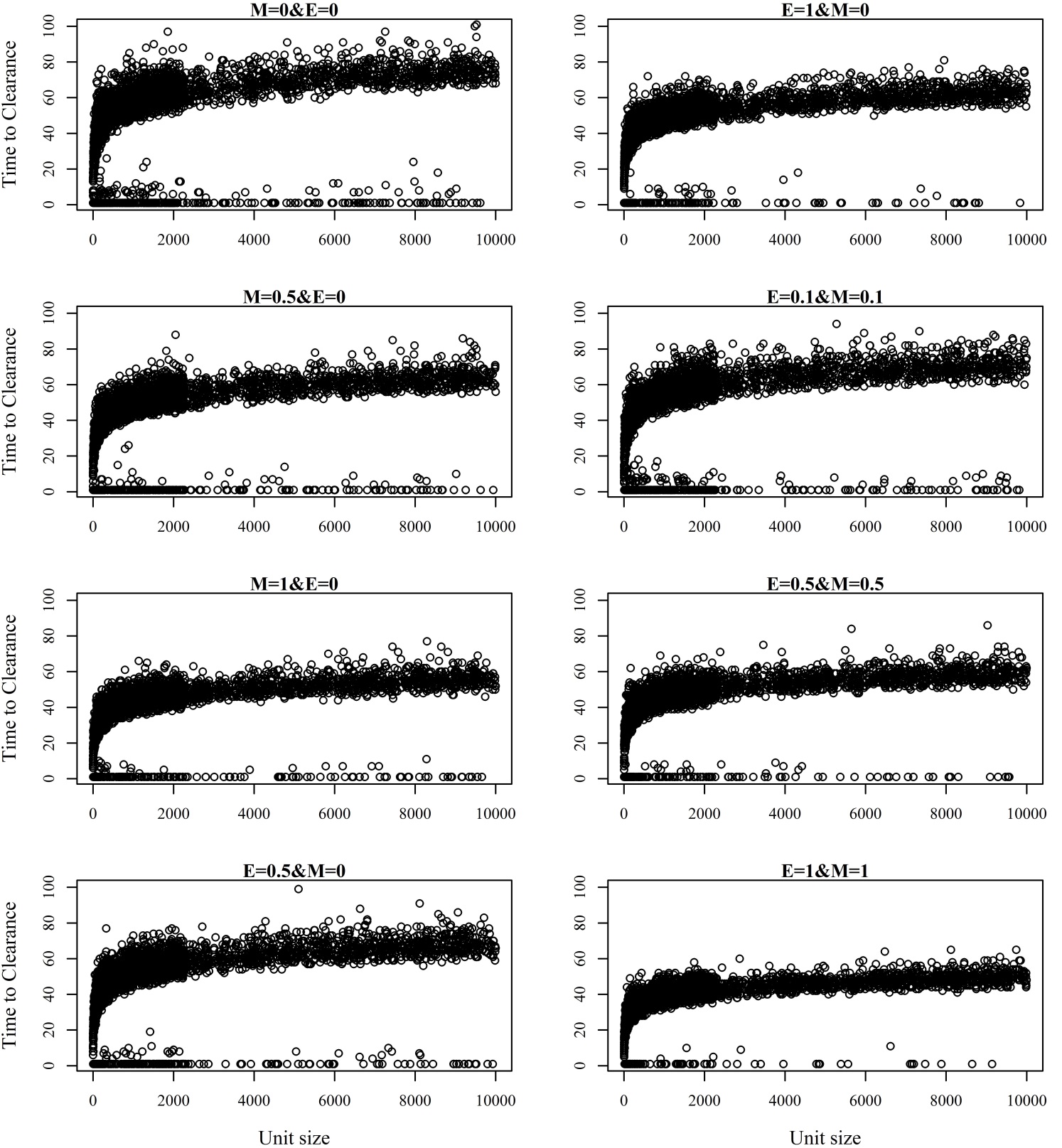


**Supplementary Figure S3b.** Association between unit size and the time until African swine fever has either died off or all animals has become infected (time to clearance - TTC) for different values μ (M) and ε (E), and with a high virus transmission rate (β = 0.6).
